# Impact of concomitant fibromyalgia on disease activity assessment and quality of life in Vietnamese patients with rheumatoid arthritis

**DOI:** 10.1093/rap/rkag067

**Published:** 2026-06-19

**Authors:** Ngan Tran Thi, Hanh Dang Thi, Hue Vu Thi, Thuy Nguyen Thi Phuong

**Affiliations:** Department of Internal Medicine, Hanoi Medical University, Hanoi, Vietnam; Department of Internal Medicine, Hanoi Medical University, Hanoi, Vietnam; Department of Internal Medicine, Hanoi Medical University, Hanoi, Vietnam; Department of Internal Medicine, Hanoi Medical University, Hanoi, Vietnam; Center for Rheumatology, Bach Mai Hospital, Hanoi, Vietnam

**Keywords:** rheumatoid arthritis, fibromyalgia, disease activity, quality of life, low- and middle-income countries

## Abstract

**Objectives:**

FM is increasingly recognised as a contributor to RA and may influence disease activity assessment. However, data from low- and middle-income countries remain limited. We aimed to investigate the prevalence and clinical impact of concomitant FM on disease activity assessment, quality of life (QoL) and treatment patterns in Vietnamese patients with RA.

**Methods:**

In this prospective cross-sectional study, adult patients with RA were assessed for FM using the 2016 ACR criteria. Disease activity [28-joint DAS with CRP (DAS28-CRP)], objective inflammatory markers, patient-reported outcomes, QoL and treatments were analysed. Multivariable linear regression analysis was performed to assess whether FM was independently associated with DAS28-CRP after adjustment for demographic and relevant clinical covariates.

**Results:**

Among 187 patients with RA, 34.2% fulfilled criteria for concomitant FM. Patients with FM had significantly higher DAS28-CRP scores compared with those without FM. The FM-related increase in DAS28-CRP was driven predominantly by subjective components, while objective inflammatory measures differed modestly. In multivariable analysis, FM remained independently associated with higher DAS28-CRP scores. Patients with FM also reported significantly worse QoL and were more likely to receive corticosteroids despite less frequent use of DMARDs.

**Conclusion:**

Concomitant FM is common among Vietnamese patients with RA and is associated with symptom amplification, poorer QoL and higher DASs independent of objective inflammation. In resource-limited settings, reliance on DAS28-CRP alone may contribute to symptom-driven treatment escalation and corticosteroid overuse. Recognition of FM is essential to support accurate disease assessment and safer, more appropriate management strategies.

Key messages

- Concomitant FM is common among Vietnamese patients with RA.
- FM inflates DAS28-CRP through subjective symptoms, independent of objective inflammation.
- In low-resource settings, DAS28 inflation may promote corticosteroid overuse and delayed DMARD optimisation.

## Introduction

RA is a chronic autoimmune disease characterized by synovial inflammation, pain and stiffness that may lead to progressive joint damage and disability if left untreated [1]. Despite substantial advances in therapeutic strategies and improved control of inflammation, many patients continue to experience significant pain, fatigue, impaired physical function and a compromised health-related quality of life (QoL). Pain is frequently interpreted by both patients and healthcare providers as a marker of ongoing inflammation, and most composite measures of RA disease activity include pain-related components such as tender joint counts (TJCs) and patient global assessment (PGA) [2]. However, accumulating evidence has demonstrated a discordance between pain and objective markers of inflammation [3].

Concomitant FM has therefore been increasingly recognized as an important contributor to persistent pain in a subset of patients with RA, including those in clinical or ultrasonographic remission or with low disease activity [4]. As a consequence, patients with FM may be less likely to achieve treatment targets and are more likely to undergo frequent switching of DMARDs. Accordingly, the EULAR has highlighted concomitant FM as an important consideration in the assessment of difficult-to-treat RA [5, 6].

The prevalence of FM is higher in patients with RA than in the general population, with reported estimates ranging from 17.7% to 29%, compared with ≈2% in the general population [7, 8]. In cross-sectional studies, the presence of FM seems to be associated with higher RA disease activity [9, 10]. Elevated levels of inflammatory cytokines in plasma and cerebrospinal fluid suggest a role for neuroinflammation and chronic systemic inflammation in the pathogenesis of FM [11, 12]. Persistent peripheral inflammation may enhance nociceptive input, while central sensitization is commonly observed in inflammatory rheumatic diseases. Considering the significant contribution of central sensitization in the pathogenesis of FM, central modulators can be accepted as the triggers of the FM process under inflammatory conditions [13].

FM is a chronic pain syndrome characterized by widespread musculoskeletal pain, fatigue, sleep disturbance and psychological distress, including anxiety and depression [14, 15]. Compared with patients with RA alone, those with concomitant FM experience poorer health-related QoL and increased healthcare utilization [15]. Given the substantial impact of FM on both physical and mental well-being, health-related QoL represents a key outcome in patients with RA. The EuroQoL five-dimension questionnaire (EQ-5D) is a widely used generic instrument for assessing health-related QoL and has been extensively applied in rheumatologic research [16]. Although previous studies have reported considerable variability in the prevalence and clinical impact of FM among patients with RA across different populations, data on the burden and consequences of FM in Vietnamese patients with RA are currently lacking. Therefore, this study aimed to investigate the prevalence of concomitant FM in Vietnamese patients with RA and to examine its impact on disease activity assessment, health-related QoL and treatment patterns in a low- and middle-income country setting.

## Methods

### Study design and patients

We performed a prospective cross-sectional study and collected data from 187 consecutive patients with RA ≥18 years of age fulfilling the 2010 ACR/EULAR classification criteria [17] and attending the Center for Rheumatology at Bach Mai Hospital, Hanoi, Vietnam, between January 2024 and April 2025. Patients were excluded if they had other causes of chronic widespread pain, including endocrinopathies (e.g. hypothyroidism), end-stage liver or kidney disease, malignancy, infection, major psychiatric disorders (such as major depressive disorders, bipolar disorder or personality disorders), metabolic bone disease, overlap with other rheumatic or connective tissue diseases or pregnancy.

All patients underwent a comprehensive medical history and physical examination at study enrolment. Data collection included demographic characteristics, clinical features, laboratory parameters and instrumental information. RA disease duration was defined as the time from RA diagnosis to study enrolment. The rheumatological assessment comprised 28-joint swollen joint counts (SJC28s), 28-joint TJCs (TJC28s) and evaluation of extra-articular manifestations. Information on relevant comorbidities, including cardiovascular diseases, diabetes mellitus, neuropsychiatric disorders, osteoporosis, osteoarthritis and FM, was systematically recorded. At the time of assessment, patients were receiving routine RA treatment, including NSAIDs, corticosteroids, conventional synthetic DMARDs (csDMARDs) and/or biologic DMARDs (bDMARDs).

RA disease activity was assessed using the 28-joint DAS based on CRP (DAS28-CRP), a composite score calculated from TJC28, SJC28, PGA and serum CRP levels [18]. The pain was assessed using a visual analogue scale (VAS). Blood samples were obtained to assess serum CRP levels and autoantibody status.

FM was diagnosed according to the 2016 ACR FM classification criteria [19]. Patients were required to meet all of the following conditions: generalized pain (defined as pain in at least four of five body regions), symptoms present at a similar level for at least 3 months and either a Widespread Pain Index (WPI) score ≥7 with a Symptom Severity Scale (SSS) score ≥5 or a WPI score of 4–6 with an SSS score ≥9.

The health-related QoL was assessed using the validated Vietnamese version of the EQ-5D [20]. The EQ-5D comprises a descriptive system and the EQ VAS. The descriptive system includes five dimensions: mobility, self-care, usual activities, pain/discomfort and anxiety/depression. Each dimension has five levels of responses in the EQ-5D-5L. Responses were converted into a single summary index (utility) ranging from 0 (death) to 1 (perfect health), but values <0 are possible and reflect health states considered worse than death. The EQ VAS records the patient’s self-rated health on a 0–10 vertical VAS.

### Ethics

The study protocol was approved by the Research Ethics Committee of Hanoi Medical University and Bach Mai Hospital (8319/QD-DHYHN). All patients provided written informed consent in accordance with the ethical standards of the 2013 version of the Declaration of Helsinki.

### Statistical analysis

Descriptive statistical analysis was used for patient characteristics and clinical variables. Continuous variables were reported as means (s.d.s) or medians [interquartile ranges (IQRs)] depending on their distribution. Categorical variables were reported as proportions and percentages. RA patients with FM and without FM were compared. Continuous variables were compared using the two-tailed *t*-test or the Mann–Whitney U-test according to their distribution. Categorical variables were compared using the Pearson chi-squared test or Fisher’s exact test. Univariable logistic regression analyses were performed to identify potential features to be included in the multivariable model. *P-*values <0.05 were considered statistically significant. All analyses were performed using the Statistical Package for the Social Sciences version 20.0 (IBM, Armonk, NY, USA).

A multivariable linear regression model was performed to assess the association between FM and disease activity DAS28-CRP, adjusting for relevant confounders. Variables were selected based on their objective representation of disease activity and/or severity; variables primarily influenced by patient perception and therefore directly related to FM were excluded. Model assumptions and influential observations were evaluated using standard diagnostic methods. Partial regression coefficients and 95% confidence intervals (95% CIs) were reported.

## Results

### Sociodemographic and clinical characteristics of the cohort

The sociodemographic characteristics of the study cohort are summarised in Table 1. A total of 187 patients with RA were included, the majority of whom were female (84.5%). The median age was 61 years (IQR 49.25–67). Only a small proportion of patients had completed college-level education (10.7%) and 67.9% were currently employed.

**Table 1:** Sociodemographic and baseline clinical characteristics of patients with RA.

Characteristics	RA patients (*n* = 187)	RA with FM (*n* = 64)	RA without FM (*n* = 123)	*P*-value
Age, years, median (IQR)	61 (49.25–67)	61.5 (54.5–67)	61 (48.75–67.0)	0.347
Female, *n* (%)	158 (84.5)	53 (82.8)	105 (85.4)	0.647
Education level, *n* (%)				0.565
≤High school	167 (89.3)	56 (87.5)	111 (90.2)	
College degree	20 (10.7)	8 (12.5)	12 (9.8)	
Marital status, *n* (%)				**0.02**
Single	10 (5.4)	2 (3.1)	8 (6.5)	
Married	163 (87.2)	52 (81.3)	111 (90.2)	
Divorced	6 (3.2)	4 (6.3)	2 (1.6)	
Widow	8 (4.3)	6 (9.4)	2 (1.6)	
Occupation, *n* (%)				**<0.001**
Housewife	16 (8.6)	8 (12.5)	8 (6.6)	
Working	127 (67.9)	31 (48.4)	96 (78.1)	
Retired	44 (23.5)	25 (39.1)	19 (15.4)	
Smoker, *n* (%)	25 (13.4)	10 (15.6)	15 (12.2)	0.513
RA disease duration, months, median (IQR)	36 (12–96)	36 (11.25–123)	42 (12.0–96.0)	0.866
Comorbidity of patients, *n* (%)	85 (45.5)	35 (54.7)	50 (40.7)	0.067
Anti-CCP positive, *n* (%)	169 (90.4)	60 (93.8)	109 (88.6)	0.307
RF positive, *n* (%)	165 (88.2)	59 (92.2)	106 (86.2)	0.18

No patients received tsDMARDs.

Significant *P-*values in bold.

The cohort was characterised by long-standing disease, with a median RA duration of 36 months (IQR 12–96) (Table 1). Most patients had active disease, as reflected by high SJC28s and TJC28s, elevated pain VAS scores and increased CRP levels. Accordingly, the mean DAS28-CRP score of 3.92 indicated overall active disease. Health-related QoL was substantially impaired, with a median EQ-5D index score of 0.672 (IQR 0.58–0.76).

Overall, 85 patients (45.5%) had at least one comorbidity, most commonly hypertension, osteoporosis, diabetes mellitus and OA. In this cohort, 48.7% of patients were receiving corticosteroid therapy. More than half of the patients (59.4%) were treated with at least one csDMARD, most commonly methotrexate, sulfasalazine or antimalarial agents as first-line therapy. In addition, 51.9% of patients were receiving bDMARDs. No patients were treated with leflunomide or targeted synthetic DMARDs.

### Comparison of patients with and without FM

In the overall cohort, 34.2% of patients (64/187) met the diagnostic criteria for FM. As shown in Table 1, there were no significant differences in age, sex, education level, RA disease duration or serological status, including RF and anti-CCP positivity, between patients who did and did not have FM. In contrast, marital and occupational status differed significantly between the two groups.

Patients with FM had significantly higher DAS28-CRP scores than those without FM [5.54 (s.d. 1.43 *vs* 3.11 (s.d. 1.24)], corresponding predominantly to high versus low disease activity, respectively (Table 2). Overall, the mean DAS28-CRP score was 2.43 points higher in the FM group. Consistently, a substantially greater proportion of patients with FM were classified as having moderate or high disease activity (92.3% *vs* 43.9%; *P* < 0.001), whereas low disease activity and remission were less frequently observed (*P* < 0.05). This difference was driven predominantly by subjective components, including significantly higher TJCs, longer reported morning stiffness and greater patient-reported pain, while differences in objective inflammatory measures, including SJCs and CRP levels, were comparatively modest. Reflecting this inflation of composite disease activity scores, patients with FM were substantially more likely to receive prednisone (76.6% *vs* 34.2%; *P* < 0.001) despite significantly less frequent use of csDMARDs (21.9% *vs* 78.9%; *P* < 0.001) and bDMARDs (37.5% *vs* 59.3%; *P* = 0.005).

**Table 2:** Clinical characteristics, disease activity and treatment patterns in RA patients with and without FM.

Characteristics	RA patients (*n* = 187)	RA with FM (*n* = 64)	RA without FM (*n* = 123)	*P*-value
Morning stiffness for ≥60 min, *n* (%)	94 (50.3)	48 (75)	46 (37.4)	**0.001**
TJC28, median (IQR)	9 (4–16)	10 (6.0–16.0)	3.5 (1.0–8.0)	**0.001**
SJC28, median (IQR)	5 (2–10)	6 (3.0–12.0)	2.5 (1.0–4.0)	**0.018**
Pain VAS (0–10), median (IQR)	5.2 (3.25–8)	6 (4.0–8.0)	3.5 (2.75–6.3)	**0.003**
PGA (0–10), median (IQR)	5 (1–8)	5 (1.0–8.0)	3 (1.0–5.0)	**0.006**
CRP, mg/l, median (IQR)	14.17 (1.65–42.6)	20.4 (5.5–73.7)	9.25 (1.225–39.9)	**0.038**
DAS28-CRP, mean (s.d.)	3.92 (1.34)	5.54 (1.43)	3.11 (1.24)	**<0.001**
Disease activity status, *n* (%)				**<0.001**
Remission	62 (33.2)	1 (1.6)	61 (49.6)	
Low disease activity	12 (6.4)	4 (6.3)	8 (6.5)	
Moderate disease activity	75 (40.1)	26 (40.6)	49 (39.8)	
High disease activity	38 (20.3)	33 (51.7)	5 (4.1)	
Current RA treatment, *n* (%)				
Prednisolone	91 (48.7)	49 (76.6)	42 (34.2)	**<0.001**
csDMARDs	111 (59.4)	14 (21.9)	97 (78.9)	**<0.001**
bDMARDs	97 (51.9)	24 (37.5)	73 (59.3)	**0.005**
tsDMARDs	0 (0)	0 (0)	0 (0)	NA

NA: not applicable (no variation between groups).

No patients received tsDMARDs.

Significant P-values in bold.

Patient-reported pain VAS and PGA scores were also significantly higher in the FM group. As shown in Table 3, patients with concomitant FM reported markedly poorer health-related QoL, with significantly lower EQ-5D index scores and a higher prevalence of problems across all EQ-5D dimensions, including mobility, self-care, usual activities, pain/discomfort and anxiety/depression.

**Table 3:** EQ-5D health-related QoL in RA patients with and without FM.

EQ-5D dimension	RA with FM (*n* = 64)	RA without FM (*n* = 123)	*P*-value
EQ-5D utility value, median (IQR)	0.572 (0.505–0.671)	0.704 (0.616–0.763)	**<0.001**
Mobility problems, *n* (%)	59 (92.2)	91 (73.9)	**0.004**
Self-care problems, *n* (%)	59 (92.2)	94 (76.4)	**0.006**
Usual activities problems, *n* (%)	62 (96.9)	102 (82.9)	**0.011**
Pain/Discomfort problems, *n* (%)	62 (96.9)	99 (80.5)	**0.008**
Anxiety/depression problems, *n* (%)	59 (92.2)	81 (65.9)	**<0.001**

Significant *P-*values in bold.

In the revised multivariable linear regression model, FM remained independently associated with higher DAS28-CRP scores, with an adjusted increase of 1.07 points (95% CI 0.64, 1.50; *P* < 0.001) (Table 4). To address potential collinearity, individual components of DAS28-CRP (including CRP and SJCs) were not included in the multivariable model. No significant associations were observed between DAS28-CRP scores and demographic factors (age and sex), disease duration, corticosteroid use or DMARD therapy in the adjusted model (all *P* > 0.05). Together, these findings suggest that elevated DAS28-CRP scores in patients with concomitant FM primarily reflect non-inflammatory symptom amplification rather than objective inflammatory disease activity, with important implications for disease assessment and treatment decisions.

**Table 4:** Multivariable linear regression model with DAS28-CRP as the dependent variable.

Independent variable	β (95% CI)	*P*-value
FM	1.073 (0.641, 1.504)	**<0.001**
Female	−0.317 (−0.764, 0.130)	0.163
Age (years)	−0.005 (−0.018, 0.007)	0.395
RA duration (months)	−0.001 (−0.002, 0.001)	0.568
Prednisolone use	−0.113 (−0.457, 0.232)	0.517
DMARD use^a^	0.07 (−0.866, 1.008)	0.882

a DMARD use includes both csDMARDs and bDMARDs.

Significant *P-*values in bold.

## Discussion

In this prospective cross-sectional study of Vietnamese patients with RA, the majority of participants were female (84.5%), which is consistent with the known female predominance in RA. This finding is also in line with previous reports from Asian populations and hospital-based cohorts [21]. Concomitant FM was common and independently associated with inflated DAS28-CRP scores and substantially poorer health-related quality of life despite only modest differences in objective inflammatory measures. These findings indicate that FM-related symptom amplification plays a major role in inflating composite DASs in RA.

FM has been shown to exert a profound impact on health status, functional capacity and quality of life [22]. It has been recently demonstrated that, in comparison with patients with RA alone, those with concomitant FM consistently report worse mental health and sleep quality, higher TJCs and pain and are significantly less likely to achieve remission or low disease activity [23]. As a consequence, composite disease activity indices may substantially overestimate inflammatory burden in patients with RA and concomitant FM. Notably, 34.2% of patients in our cohort met the diagnostic criteria for FM, a prevalence comparable to that reported in previous studies [13, 24]. More recently, Joharatnam *et al.* [25] demonstrated that 48% of their patients with established RA satisfied the same criteria for FM. To our knowledge, this is the first study to estimate the prevalence of FM among Vietnamese patients with RA, highlighting a considerable and previously underrecognized burden in this population. Our findings are also broadly consistent with emerging data from Asian populations. Recent studies from India have reported FM prevalence rates ranging from ≈30–40% among patients with RA, with similarly higher DASs and poorer patient-reported outcomes in patients with concomitant FM [24]. Similar findings have been reported in Bangladesh and other Asian cohorts suggest that FM substantially influences composite disease activity assessment across diverse healthcare settings [26, 27]. Nevertheless, data from Southeast Asia remain limited, and our study therefore provides important evidence from a Vietnamese rheumatology population.

We found that concomitant FM was independently associated with higher DAS28-CRP scores and substantially poorer health-related QoL, as reflected by lower EQ-5D utility values. Despite advances in RA management, patients with FM reported significantly worse QoL across all EQ-5D dimensions, consistent with previous reports demonstrating the additive burden of FM on both physical and mental health outcomes in RA [28–30]. Although FM is not classically associated with inflammatory synovitis, stiffness is a commonly reported symptom in patients with FM. However, in contrast to inflammatory arthritis, FM-related stiffness is not driven by synovial inflammation and typically does not demonstrate the same diurnal improvement pattern [31]. Therefore, the longer duration of morning stiffness observed in our cohort may reflect central sensitisation and heightened symptom perception rather than inflammatory activity. The differences in marital and occupational status between patients with and without FM further reflect the broader psychosocial and functional impact of FM, which is also captured by worse EQ-5D anxiety/depression scores. Notably, the marked impairment in the anxiety/depression dimension suggests that psychological distress may act as a key mediator linking FM-related symptom amplification to higher patient-reported disease activity. Together, these findings highlight the close association between symptom burden, mental well-being and social functioning in patients with RA and concomitant FM.

In the present study, patients with concomitant FM had significantly higher DAS28-CRP scores than those with RA alone, consistent with previous reports [9, 32, 33]. Analysis of individual DAS28 components showed that this difference was largely driven by subjective measures, particularly TJCs, patient-reported pain and PGA, whereas differences in objective inflammatory markers, including SJCs and CRP levels, were comparatively modest. These findings support prior observations that higher DAS28 scores in patients with FM is explained primarily by increased TJC and symptom burden, which may lead to overestimation of inflammatory disease activity in RA and misclassification of disease activity status [9, 25, 34]. Greater pain contributes to a higher DAS28 score and a greater sensitivity to pain is usually associated with psychological distress, with a consequent worsening of general health status [31, 32, 35].

Higher PGA scores and TJCs in patients with FM may reflect central sensitisation, a core pathophysiological feature of FM characterised by amplified pain processing and generalised hyperalgesia [3, 36]. Although modest differences in SJCs and CRP levels were observed, these were insufficient to explain the pronounced differences in DAS28-CRP, suggesting that subjective components are the primary drivers of score elevation.

Taken together, these mechanistic considerations support the notion that the observed differences in DAS28-CRP are largely driven by non-inflammatory symptom amplification rather than objective inflammatory activity. No significant between-group differences were observed in disease duration or serological status, including RF and anti-CCP positivity. Collectively, these findings indicate that the pronounced elevation in DAS28-CRP among patients with FM predominantly reflects FM-related symptom burden rather than unequivocal differences in inflammatory disease activity. Nevertheless, a modest increase in inflammatory burden cannot be entirely excluded, particularly in light of the more frequent use of corticosteroids among patients with FM.

This interpretation is further supported by the multivariable linear regression analysis (Table 4). After adjustment for demographic and clinical covariates, FM remained independently associated with a clinically and statistically significant increase in DAS28-CRP. In contrast, demographic factors, disease duration, corticosteroid use and DMARD therapy were not significant predictors in the adjusted model. Together, these findings underscore that FM independently inflates composite DASs, beyond what can be explained by measurable inflammation.

Taken together, the combined physical, psychological and social burden associated with concomitant FM may complicate the clinical assessment of disease activity and help explain observed differences in treatment. Treatment patterns observed in this study further underscore this challenge. In our cohort, patients with FM were more likely to receive corticosteroids, whereas both csDMARD and bDMARD use was less frequent compared with patients without FM. The overall proportion of patients receiving csDMARDs or bDMARDs in this cohort was relatively low, which may reflect real-world treatment patterns in a resource-limited setting, where access to advanced therapies, particularly biologics, remains constrained. In addition, treatment initiation or optimisation may be delayed in some patients, with greater reliance on symptomatic therapies such as corticosteroids. This pattern likely reflects the challenge of distinguishing inflammatory disease activity from FM-related symptoms in routine practice, where pain, tenderness, fatigue and prolonged morning stiffness may be interpreted as persistent inflammatory activity. Such symptom-driven management may contribute to increased corticosteroid exposure while potentially limiting timely escalation or optimisation of disease-modifying therapy. In settings where treatment decisions rely heavily on composite indices such as DAS28, the presence of FM may therefore increase the risk of overtreatment with corticosteroids and delayed adjustment of DMARD therapy. This issue may be particularly relevant in low- and middle-income countries, where access to advanced imaging and biologic agents is limited and treatment decisions are often guided predominantly by clinical indices. Accordingly, the potential inflation of DAS28 by subjective components in the context of limited or absent objective inflammation underscores the importance of careful clinical interpretation of composite DASs [37]. Recognition of concomitant FM is essential to support more accurate assessment of disease activity and to inform appropriate, individualised treatment decisions in patients with RA.

Given that DAS28 remains a cornerstone outcome measure in both clinical practice and clinical trials, the impact of concomitant FM on its interpretation has important clinical and research implications [33]. Our findings support previous observations and current EULAR recommendations identifying FM as a key contributor to difficult-to-treat RA, particularly in patients with persistently high DAS28-CRP despite limited objective inflammation [38]. These results underscore the need for cautious interpretation of composite disease activity indices in patients with RA and FM and support the use of comprehensive clinical judgment incorporating objective inflammatory markers with assessment of pain mechanisms.

This study demonstrates that concomitant FM is common among Vietnamese patients with RA attending tertiary rheumatology care and is associated with higher DAS28-CRP scores and poorer health-related QoL. The FM-related increase in DAS28 appears to be driven predominantly by subjective components rather than objective inflammatory activity, raising the risk of clinically meaningful misclassification of disease activity. Consequently, reliance on DAS28-CRP alone in patients with concomitant FM may overestimate inflammatory burden and lead to inappropriate treatment escalation and thus is essential to support more accurate disease assessment and informed clinical decision making.

While these findings provide important insights into the impact of concomitant FM on disease activity assessment and QoL in Vietnamese patients with RA, several limitations should be acknowledged. The cross-sectional design precludes assessment of temporal or causal relationships between FM and RA disease activity, and the timing of FM onset relative to RA diagnosis or treatment initiation could not be determined. In addition, this single-centre study was conducted at a major tertiary referral hospital, which may have enriched the cohort for patients with more severe or treatment-refractory disease, potentially contributing to the relatively high prevalence of FM and limiting generalisability to RA populations managed in primary or secondary care. Finally, advanced imaging was not systematically performed to evaluate subclinical synovitis, which may have provided further insights into the discordance between subjective symptoms and objective inflammatory activity.

Despite these limitations, this study has several important strengths. We applied contemporary ACR classification criteria for both RA and FM and conducted a comprehensive assessment of clinical, laboratory and patient-reported outcomes. Importantly, this study provides the first data on the prevalence and clinical impact of concomitant FM in Vietnamese patients with RA, an underrepresented population in the rheumatology literature. Collectively, these findings reinforce the need for cautious interpretation of composite disease activity measures in routine practice, particularly in patients with persistent pain despite limited objective inflammation.

## Data Availability

The data are available from the corresponding author.
